# Effectiveness of Different Rituximab Doses Combined with Leflunomide in the Treatment or Retreatment of Rheumatoid Arthritis: Part 2 of a Randomized, Placebo-Controlled, Investigator-Initiated Clinical Trial (AMARA)

**DOI:** 10.3390/jcm11247316

**Published:** 2022-12-09

**Authors:** Michaela Koehm, Ann C. Foldenauer, Tanja Rossmanith, Rieke Alten, Martin Aringer, Marina Backhaus, Gerd R. Burmester, Eugen Feist, Herbert Kellner, Klaus Krueger, Ulf Müller-Ladner, Andrea Rubbert-Roth, Hans-Peter Tony, Siegfried Wassenberg, Harald Burkhardt, Frank Behrens

**Affiliations:** 1 Fraunhofer Institute for Molecular Biology and Applied Ecology IME, Branch for Translational Medicine & Pharmacology ITMP, 60596 Frankfurt am Main, Germany; 2 Division of Rheumatology, University Hospital Frankfurt, Goethe University, 60596 Frankfurt am Main, Germany; 3 Fraunhofer Cluster of Excellence Immune-Mediated Diseases (CIMD), 60596 Frankfurt am Main, Germany; 4 Schlosspark-Klinik, 14059 Berlin, Germany; 5 Department of Medicine III, University Medical Centre, Faculty of Medicine, Dresden University of Technology, 01307 Dresden, Germany; 6 Park-Klinik Weissensee, 13086 Berlin, Germany; 7 Department of Rheumatology and Clinical Immunology, Charité–Universitätsmedizin Berlin, Freie Universität Berlin and Humboldt-Universität zu Berlin, 10117 Berlin, Germany; 8 Praxis für Rheumatologie, 80639 Munich, Germany; 9 Praxiszentrum St. Bonifatius, 81541 Munich, Germany; 10 Department of Rheumatology and Clinical Immunology, Justus-Liebig University, Campus Kerckhoff, 61231 Bad Nauheim, Germany; 11 Division of Rheumatology and Clinical Immunology, Cantonal Hospital St. Gallen, 9007 St. Gallen, Switzerland; 12 Department of Rheumatology/Immunology, University of Würzburg, 97080 Würzburg, Germany; 13 Rheumazentrum Ratingen, 40878 Ratingen, Germany

**Keywords:** rheumatoid arthritis, rituximab, dosing, leflunomide, clinical trial

## Abstract

Background: The optimal dose of rituximab in combination with leflunomide in patients with rheumatoid arthritis (RA) is not known. Methods: In Part 1 (previously reported) of the investigator-initiated AMARA study (EudraCT 2009-015950-39; ClinicalTrials.gov NCT01244958), improvements at week (W)24 were observed in patients randomized to rituximab + leflunomide compared with placebo + leflunomide. In the study reported here (Part 2), Part 1 responders received rituximab 500 or 1000 mg at W24/26 plus ongoing leflunomide. Patients were randomized at baseline to their eventual W24 treatment group. The Part 2 primary outcome was the mean Disease Activity Score-28 joints (DAS28) at W52, based on the last observation carried forward (LOCF) analyses and a two-sided analysis of variance. Patient-reported outcomes (PROs) and adverse events were evaluated. Results: Eighty-three patients received rituximab at W24/26 (31 rituximab→rituximab 1000 mg; 29 rituximab→rituximab 500 mg; 10 placebo→rituximab 1000 mg; 13 placebo→rituximab 500 mg). At W52, there were no significant differences in DAS28 between rituximab doses in patients originally treated with rituximab or those originally treated with placebo. In the Part 1 placebo group, the higher rituximab dose was associated with greater improvements in ACR response rates and some PROs. Adverse events were similar regardless of rituximab dose. Conclusions: Retreatment with rituximab 500 mg and 1000 mg showed comparable efficacy, whereas an initial dose of rituximab 500 mg was associated with lower response rates versus 1000 mg. Reduced treatment response with the lower dose in patients initially treated with placebo may have been influenced by small numbers and baseline disease activity.

## 1. Introduction

Therapy with the B-cell depleting agent rituximab is a valuable treatment option in rheumatoid arthritis (RA) [1,2,3], but questions remain concerning optimal dosing of initial and retreatment strategies. For initial treatment, both the European Medicines Agency (EMA) summary of product characteristics [4] and the US Food and Drug Administration (FDA) prescribing information [5] state that rituximab should be administered as two 1000 mg IV infusions given 2 weeks apart to patients being treated with methotrexate. However, some data suggest that initial treatment with 2 × 500 mg doses of rituximab results in clinical outcomes equivalent to treatment with 2 × 1000 doses of rituximab [2,6,7,8]. The situation with rituximab retreatment is even more ambiguous, as neither the EMA nor the FDA prescribing information provides specific guidance on this point [4,5]. Despite several studies [9,10,11,12], including the multicentre, double-blinded, phase 3 MIRROR trial [13], there is still no definitive answer on the best dose for rituximab retreatment in patients with RA [14]. In routine clinical care, the dosage of 1000 mg IV given two weeks apart is often used as retreatment, but the use of a potentially higher than necessary dose could increase the risk of adverse reactions and alter risk-benefit considerations. For both initial treatment and retreatment, most experience to date has involved regimens consisting of rituximab plus methotrexate; the effect of different rituximab doses in patients being treated with concomitant leflunomide has not been rigorously evaluated.

As previously reported [15], we investigated the efficacy and safety of rituximab + leflunomide compared with placebo + leflunomide in patients with active RA and an inadequate response to leflunomide. In the rituximab arm, patients received the approved standard induction dose of rituximab (two doses of rituximab 1000 mg two weeks apart). Here we investigate the clinical response to two different rituximab dosing schedules, 2 × 500 mg and 2 × 1000 mg, administered 24 and 26 weeks after the initial rituximab dose as retreatment and 24 weeks after the first placebo dose as initial treatment, in patients with RA receiving ongoing concomitant leflunomide. The objective of this study was to determine the optimal rituximab dose when used in combination with leflunomide to treat patients with RA.

## 2. Patients and Methods

### 2.1. Study Design

The Addition of MabThera to Arava in the RA (AMARA) study was an investigator-initiated prospective, randomized, double-blind, placebo-controlled, phase 3 clinical trial conducted at 33 clinical centers in Germany between 8 August 2010 and 28 January 2015 (Supplementary Section S1) [15]. The AMARA study was registered with the European Union Drug Regulating Authorities Clinical Trials Database (EudraCT 2009-015950-39) on 28 December 2009, prior to submission to ethical committees and to inclusion of the first subject; subsequently it was additionally registered with ClinicalTrials.gov (NCT01244958) to provide broader access to the protocol. The study protocol was approved by the ethics committee of Goethe University (Ethikkommission des Fachbereichs Medizin der Goethe Universität) and by local ethics committees at participating clinical sites. The study was conducted in accordance with the Declaration of Helsinki. All patients gave written informed consent for study participation.

The AMARA study design for Part 1 has been reported previously [15]. Briefly, adult patients with RA and an inadequate response to leflunomide were randomized 2:1 to 1000 mg rituximab or placebo administered as intravenous infusions on day 1 and day 15 by use of a computer-prepared randomization list. All patients remained on their stable doses of leflunomide (10–20 mg/day). Patients who did not respond to therapy by week 16, as defined by Disease Activity Score-28 joints based on the erythrocyte sedimentation rate (DAS28-ESR) change from baseline <0.6 or <20% improvement in both tender and swollen joints, were considered non-responders and were offered rescue therapy (standard of care). These patients did not continue into Part 2 of the study. The primary efficacy outcome for Part 1 was the difference in the American College of Rheumatology (ACR) 50% improvement responses at week 24 [15].

In Part 2 of the study, all patients, including patients who were in the placebo arm group during Part 1, received rituximab 500 mg or 1000 mg at week 24 and week 26 (a given patient received the same dose at both visits). Patients were randomized at baseline to their eventual week 24 treatment group in order to prevent a selection bias for patients who remained in the study. Both patients and investigators were blinded to the rituximab dose. Rituximab was administered under the supervision of study staff at the clinical center. During Parts 1 and 2, all patients continued stable oral leflunomide treatment at the pre-enrollment dose (10 to 20 mg/day). Adherence to leflunomide therapy was not formally assessed, but its use as concomitant therapy at a stable dose was verified at each visit. Patients were allowed to continue corticosteroid therapy (≤10 mg/day prednisone or equivalent) and oral non-steroidal anti-inflammatory drugs at a stable dose.

### 2.2. Outcomes

The primary efficacy outcome for AMARA Part 2 was mean DAS28 (based on erythrocyte sedimentation rate [ESR]) at week 52. Secondary efficacy outcomes included mean DAS28 at other timepoints between week 24 and week 52, and ACR20, ACR50, and ACR70 response rates from baseline (week 0). Patient-reported outcomes included Short-Form 36 (SF-36) subscales (higher scores indicated better status), patient global assessments (PtGA) on a 100-mm visual analogue scale (VAS) (lower scores indicate less disease impact), Health Assessment Questionnaire-Disability Index (HAQ-DI) (lower scores indicated better function), and Functional Assessment of Chronic Illness Therapy-Fatigue (FACIT-F) (higher scores indicated less fatigue).

Safety analyses were based on reports of adverse events (AEs) and serious AEs (SAEs) classified by MedDRA system organ class (SOC) and preferred term. Peripheral CD19+/CD20+ cells were measured by fluorescence-activated cell sorting to evaluate rituximab-associated B-cell depletion.

### 2.3. Statistical Analysis

Sample size calculations for AMARA Part 1 have been reported previously (14). AMARA Part 2 was exploratory and so was not statistically powered for conclusive analyses. Efficacy analyses were conducted on the intention-to-treat (ITT) population, defined as all patients who received at least one dose of study medication and had at least one assessment under study medication, with imputation using the last observation carried forward (LOCF) for the primary and secondary outcomes. To compare all four treatment arms in Part 2, the primary efficacy outcome of Part 2 (DAS28-ESR) was analyzed by analysis of variance (ANOVA), and ACR rates were analyzed by Fisher’s test (2-tail) at different visits using the stepdown Bonferroni method for adjusting P values (post hoc analysis). *p* values < 0.05 were considered statistically significant.

## 3. Results

### 3.1. Patient Disposition

**Figure 1:**
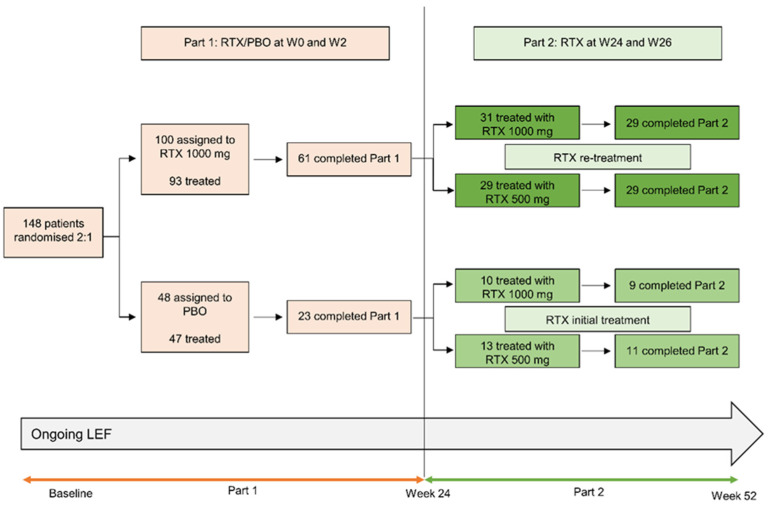
Patient disposition in part 2 of the AMARA study. Randomizations to re-treatment groups occurred at baseline. LEF, leflunomide; PBO, placebo; RTX, rituximab; W, week.

Of 140 leflunomide-treated patients randomized to treatment with rituximab or placebo between 13 August 2010 and 28 January 2015, 84 completed Part 1 of the trial and 83 received a dose of rituximab in Part 2 at week 24 and were included in LOCF analyses (Figure 1). At baseline (prior to entry into Part 1), characteristics of demographic and disease characteristics of patients were generally well-matched, although lower numbers of rheumatoid factor seropositive patients and a longer disease duration were noted in patients who were randomized to initial placebo followed by 1000 mg rituximab (Table 1). Disease characteristics at week 24 were more favorable in patients who received rituximab in Part 1 (Table 1), although all groups showed improvements from baseline.

Of the 83 patients who entered Part 2, 78 patients (94.0%) completed the study and had data at week 52 (Figure 1). Reasons for study discontinuation in patients originally treated with rituximab were withdrawal of consent (*n* = 1) and administrative reasons (*n* = 1) (both in the rituximab 1000 mg arm). Reasons for study discontinuation in patients originally treated with placebo were adverse events (*n* = 2; rituximab 500 mg arm) and other reasons (*n* = 1; rituximab 1000 mg arm).

### 3.2. DAS28 Outcomes

**Figure 2:**
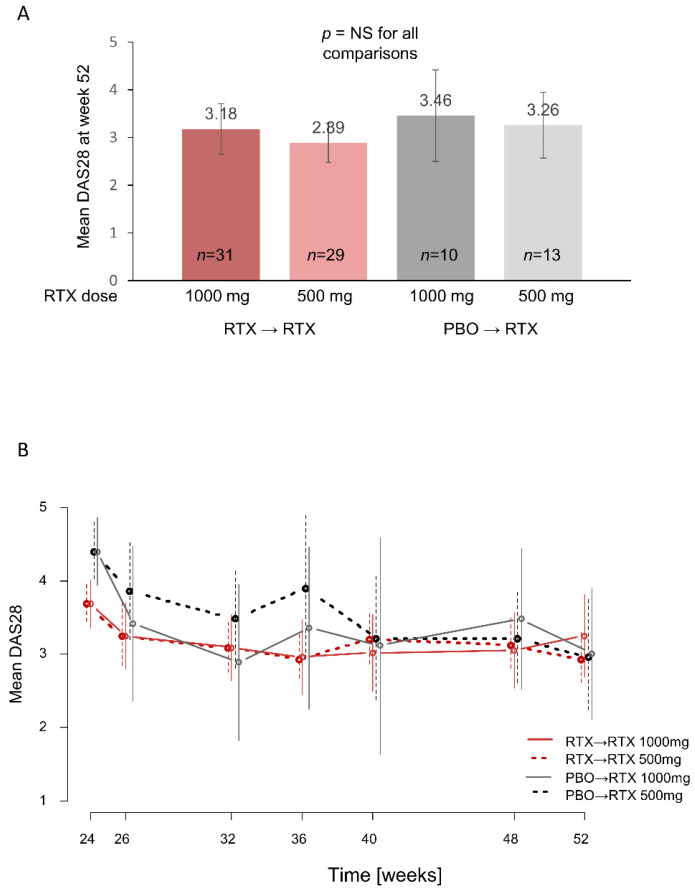
DAS28 outcomes during Part 2 of the AMARA study. (**A**) Mean DAS28 at week 52 in LOCF analyses. (**B**) Mean DAS28 over time from week 24 to week 52. For both (**A**) and (**B**), vertical lines indicate 95% CI. DAS28, Disease Activity Score based on 28 joints; NS, not significant; PBO, placebo; RTX, rituximab.

At week 52, the primary endpoint for Part 2 of the study, there were no significant differences in mean DAS28 between the rituximab 1000 mg and 500 mg groups in patients originally treated with rituximab (mean DAS28 of 3.18 [95% confidence interval (CI) 2.65–3.71] for rituximab 1000 mg vs. 2.89 [95% CI 2.48–3.30] for rituximab 500 mg; *p* > 0.05) or in patients originally treated with placebo (3.46 [95% CI 2.50–4.41] vs. 3.26 [95% CI 2.57–3.94]; ANOVA *p* = 0.59, indicating that none of the pairwise comparisons showed a significant difference) (Figure 2A). Changes in mean DAS28 from baseline to week 52 and from week 24 to week 52 were also comparable in all groups. In evaluations of change in DAS28 over time, patients in the placebo arm in Part 1 appeared to achieve a faster reduction in DAS28 with the higher rituximab dose, but by week 40 mean DAS28 values were comparable between the low- and high-dose groups (Figure 2B). Patients who were in the rituximab arm in Part 1 had similar responses to rituximab retreatment regardless of the dose.

### 3.3. ACR Response Rates

**Figure 3:**
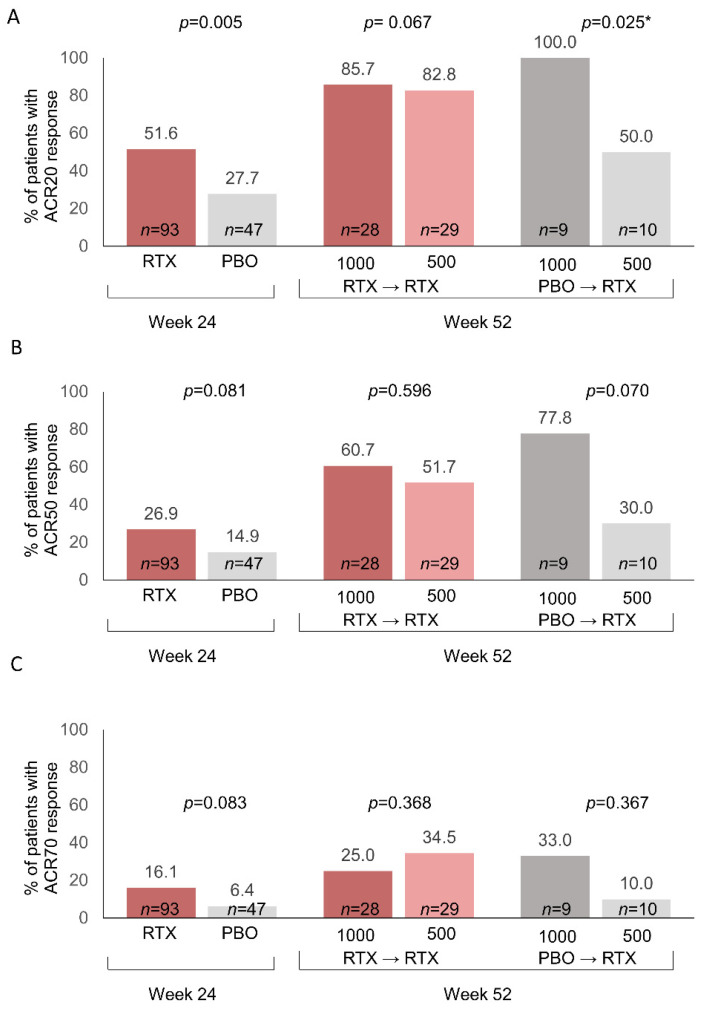
Percent of patients with ACR responses following rituximab treatment/retreatment based on observed data. (**A**) ACR20 responses; (**B**) ACR50 responses; (**C**) ACR70 responses. Data for week 52 are based on the number of patients with ACR responses divided by the number of patients with ACR data. Fisher’s exact *p*-values (2-tail) are shown. Week 52 data were missing for 3 patients in the RTX→RTX 2 × 1000 mg group, 1 patient in the PBO→RTX 2 × 1000 mg group, and 3 patients in the PBO→RTX 2 × 500 mg group. * Stepdown Bonferroni adjusted *p* value = 0.051. ACR, American College of Rheumatology; PBO, placebo; RTX, rituximab.

Analyses of the proportion of patients achieving ACR response criteria from baseline (week 0) indicated that the two doses of rituximab resulted in similar outcomes in patients who had been previously treated with rituximab 1000 mg during Part 1 of the study (Figure 3). In patients who were treated with placebo in Part 1, the 1000 mg dose of rituximab as initial treatment resulted in significantly higher ACR20 response rates (*p* = 0.025) and numerically higher ACR50 and ACR70 response rates, compared with the 500 mg dose (Figure 3). Discrepancies in those outcomes could also be seen in the pre-randomized groups at week 24 prior to rituximab treatment (Table 1). We therefore performed a post hoc analysis of adjusted *p* values using the stepdown Bonferroni method and determined that all adjusted *p* values were nonsignificant, although the comparison of ACR20 rates in the placebo→rituximab 1000 mg vs. 500 mg dose approached significance (*p* = 0.051).

### 3.4. Patient-reported Outcomes

**Figure 4:**
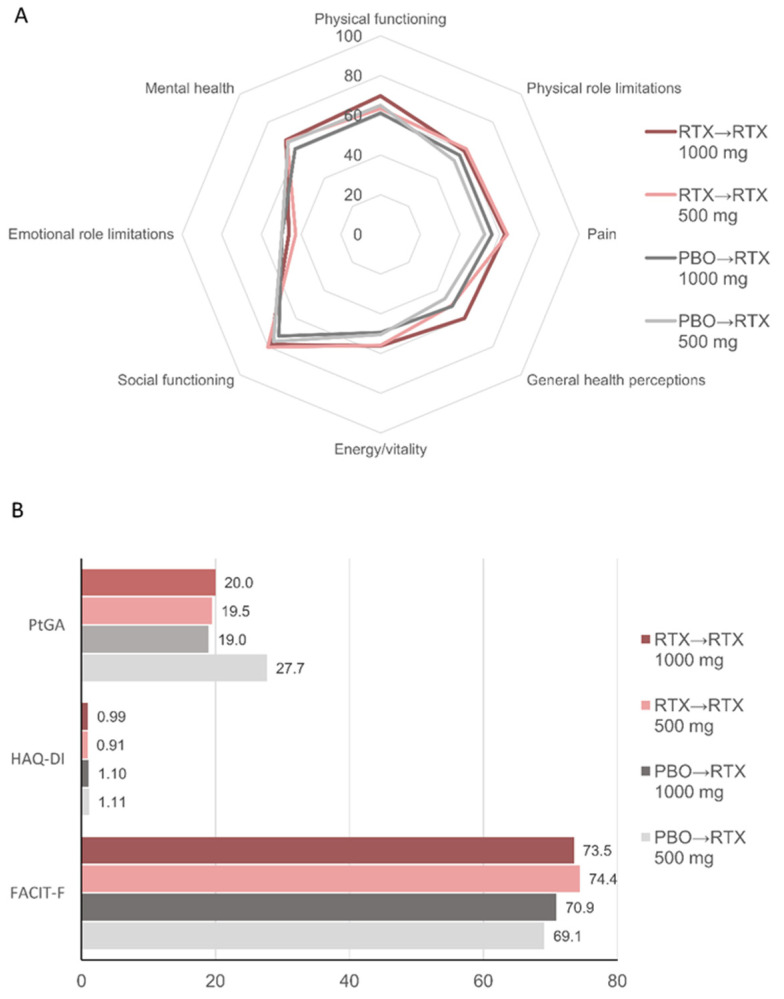
Patient-reported outcomes at week 52. Mean values for (**A**) SF-36 domains; (**B**) Patient global assessment (PtGA), Health Assessment Questionnaire-Disability Index (HAQ-DI), and FACIT-fatigue (FACIT-F) scores. For SF-36 and FACIT-F, higher scores indicate better status. For PtGA and HAQ-DI, lower scores indicate better status. PBO, placebo; RTX, rituximab.

PRO values at week 24 were consistent with the overall more favorable health status in patients who had received rituximab during Part 1 of the study compared with patients who had received placebo (Table 1). At week 52, PRO values were comparable across the four subgroups (Figure 4). In patients treated with placebo during Part 1, some outcomes, including the SF-36 general health perceptions and PtGA, were more favorable in patients who received treatment with rituximab 1000 mg compared with those receiving the lower dose. As with ACR response rates, discrepancies in those outcomes could also be seen at week 24 prior to rituximab treatment (Table 1).

### 3.5. Safety

Our analyses of AEs during Part 1 found a generally good tolerability profile for rituximab + leflunomide compared with placebo + leflunomide, but the rituximab group had higher rates of SAEs (20.4% vs. 2.1%), mostly involving infections and musculoskeletal disorders [15]. During Part 2, AEs were comparable among subgroups, although patients initially treated with rituximab appeared to have higher rates of infections compared with patients initially treated with placebo (Table 2). SAEs occurred in 16/83 (19.3%) of patients overall and in 13/60 (21.7%) of patients receiving rituximab retreatment compared with 4/23 (17.4%) of patients receiving initial rituximab treatment following treatment with placebo in Part 1 (Table 2). Higher doses of rituximab were not associated with higher SAE rates: the rate of SAEs in the rituximab 500 mg group was 25% (10/40) compared with 14.6% (6/41) for the 1000 mg dose. Surgical and medical procedures were the most common SAEs (five patients, including two intervertebral disc operations), followed by infections (four patients; abdominal wall abscess, respiratory tract infection, gastroenteritis, and erysipelas) and musculoskeletal and connective tissue disorders (four patients). All SAEs resolved successfully; one (bunion surgery) resolved with sequelae.

Assessments of changes in B cell counts from week 24 to week 52 showed continued reductions in B cell levels in patients receiving retreatment with rituximab, and a rapid reduction in B cell counts between weeks 24 and 26 in patients receiving initial treatment with rituximab (Supplementary Figure S1). There were no clear differences in B cell reductions among the different rituximab dosage groups. However, greater increases in B cell counts after week 40 were observed in patients receiving the 500 mg dose compared with the 1000 mg dose.

## 4. Discussion

Data regarding the optimal dose of rituximab in patients with RA are still scarce, and appropriate dosing has not been evaluated thus far in patients treated with concomitant leflunomide instead of MTX. Lower doses of rituximab have the potential to reduce costs and improve safety. As part of an investigator-initiated clinical trial of the combination of rituximab + leflunomide, we evaluated rituximab dosing schedules of 2 × 500 mg and 2 × 1000 mg as retreatment following previous rituximab treatment or as newly initiated rituximab therapy in patients with RA receiving treatment with leflunomide. We found that in patients retreated with rituximab, the lower rituximab dose was associated with clinical outcomes comparable to the higher (1000 mg) dose. For patients on leflunomide therapy receiving initial rituximab treatment (placebo patients during Part 1), dosing with 1000 mg rituximab resulted in similar mean DAS28 at week 52 compared with the 500 mg dose, but the higher dose was associated with a faster decrease in DAS28 scores and better ACR response rates and PROs. However, clear differences between the two placebo subgroups in ACR responses and PROs were also observed at week 24 prior to initiation of rituximab treatment. It is therefore possible that these differences were not driven by the rituximab dose but by patient characteristics in these smaller subgroups. The comparability of the 500 and 1000 mg rituximab doses as initial therapy is consistent with observational data [6,7] and with phase IIb and phase III trials in patients treated with rituximab plus MTX [2,8,13], while data also support the comparability of these doses as retreatment in patients receiving concomitant therapy with MTX [13] or other conventional synthetic disease-modifying antirheumatic drugs (DMARDs) [12].

However, the longer maintenance of B cell depletion, which occurred in both the retreatment and initial treatment groups, was compatible with prolonged therapeutic benefit. Increases in B cell counts were not accompanied by worsening in DAS28 over the course of our study. Nevertheless, the diminished maintenance of B cell depletion suggests that the clinical status of patients on lower doses of rituximab should be closely monitored near the end of the dosing interval.

Overall, the combination of rituximab + leflunomide was well tolerated. Although about half of patients reported an infection or infestation, these were rarely severe. Other biological DMARDs also increase the risk of infection [16], and our safety data are consistent with other studies in which the risk for serious infection with rituximab was similar to or lower than the infection risks of other biological DMARDs [17]. A five-year observational study of rituximab in RA found that neither the number of rituximab courses nor the time from the first rituximab dose had a significant impact on the rate of infections [18].

We did not observe a clear dose-dependent effect on AE rates. Other studies with varying dosing regimens have differed on the safety profile of higher versus lower dose rituximab. Although overall AEs with different rituximab doses have been largely similar [9,10,11,12,13], some studies have reported higher infection rates with higher rituximab doses [10,11,12], while others have found lower infection rates with higher doses [9,13].

Our study has several limitations, most notably the small numbers of patients in the groups treated with placebo in Part 1 (Part 2 placebo→rituximab groups). Differences in leflunomide doses or concomitant therapy with corticosteroids or anti-inflammatory drugs could potentially have influenced outcomes and confounded the analyses. Because the primary objective was to evaluate short-term clinical results, no analyses were performed on longer-term outcomes such as radiographic progression. It is possible that the advantages of higher rituximab doses may require more extended evaluations. A two-year study found that long-term radiographic outcomes were improved in patients treated with rituximab 2 × 1000 mg vs. 2 × 500 mg, although variability in treatment doses and number of courses could have confounded this finding [19]. Longer-term studies with larger numbers of patients will be required to address this question.

## 5. Conclusions

In conclusion, retreatment with two doses of rituximab at either 500 mg or 1000 mg appears to result in comparable short-term clinical outcomes in patients with RA receiving leflunomide therapy. The data are less clear with regards to initial treatment. For patients on leflunomide who received initial treatment with rituximab at week 24, the 1000 mg dose was associated with higher ACR response rates and improvements in some PROs at week 52. These findings may indicate that patients with severe disease or those who require rapid disease control may benefit from initial treatment with the higher rituximab dose. However, it is possible these differences in outcomes for the 1000 mg vs. 500 mg rituximab dose in patients receiving initial treatment were due to confounding factors given the similar trends observed at week 24, prior to rituximab treatment. No unexpected adverse events were observed, but clinicians managing patients treated with rituximab + leflunomide should be aware of the potential risk of infection SAEs. Together, data from Part 1 and Part 2 of the AMARA trial have demonstrated the feasibility of combining leflunomide with rituximab in patients with RA and support the use of a reduced rituximab dose (2 × 500 mg) for retreatment in patients with a favorable response to this combination therapy.

## Figures and Tables

**Table 1:** Baseline and week 24 patient and disease characteristics for patients entering Part 2 of the AMARA study. Data are presented as mean (standard deviation) unless otherwise indicated.

Characteristic	Rituximab→Rituximab		Placebo→Rituximab	
	Rituximab 1000 mg	Rituximab 500 mg	Rituximab 1000 mg	Rituximab 500 mg
*n*	31	29	10	13
Baseline				
Age, years	58.2 (11.3)	57.1 (12.9)	54.7 (6.3)	55.3 (11.0)
BMI, kg/m^2^	26.8 (5.3)	25.9 (6.1)	29.8 (5.0)	25.9 (6.0)
Females, *n* (%)	25 (80.7%)	19 (65.5%)	7 (70.0%)	9 (69.2%)
RF seropositive, *n* (%)	21 (67.7%)	19 (65.5%)	4 (40.0%)	7 (53.9%)
Anti-CCP seropositive,^a^ *n* (%)	22 (71.0%)	18 (62.1%)	4 (40.0%)	7 (53.9%)
Previous use of conventional DMARDs (%)	30 (96.8%)	17 (93.8%)	10 (100.0%)	12 (94.6%)
Patients with at least one previous anti-TNF therapy, *n* (%)	1 (3.2%)	4 (13.8%)	0	1 (7.7%)
Corticosteroid dose, ^b,c^ mean (SD) [median; Q1, Q3]	6.3 (2.7) [5.0; 2.0, 10.0]	5.3 (1.7) [5.0; 2.0, 10.0]	6.7 (2.9) [5.0; 5.0, 10.0]	5.5 (1.1) [5.0; 5.0, 7.5]
Leflunomide dose, ^c^ mean (SD)	17.2 (4.5)	19.6 (2.0)	19.0 (3.2)	18.3 (3.9)
10 mg dose, *n* (%)	8 (25.8%)	1 (4.4%)	1 (10.0%)	2 (15.4%)
20 mg dose, *n* (%)	23 (74.2%)	28 (96.6%)	9 (90.0%)	11 (84.6%)
Disease duration, years, mean (SD) [median; Q1, Q3]	8.0 (9.7) [4.4; 1.9, 11.5]	7.4 (7.6) [3.8; 2.0, 12.5]	10.6 (12.8) [5.1; 4.1, 13.8]	4.5 (3.7) [3.1; 2.2, 4.7]
DAS 28	5.7 (1.1)	5.3 (1.0)	5.9 (1.0)	5.3 (1.0)
CDAI, mean (SD) [median; Q1, Q3]	31.2 (11.8) [28.7; 23.7, 39.2]	27.4 (8.7) [28.2; 21.0, 34.0]	36.4 (12.7) [32.5; 27.2, 51.2]	31.8 (10.5) [28.2; 24.4, 35.5]
Tender joint count (28 joints)	10.5 (5.8)	9.4 (4.2)	14.0 (6.9)	13.3 (6.3)
Swollen joint count (28 joints)	8.7 (4.3)	7.8 (3.5)	10.5 (5.8)	7.3 (2.9)
Tender joint count (68 joints)	15.5 (10.4)	13.9 (6.8)	20.9 (12.9)	19.0 (11.0)
Swollen joint count 66 joints)	10.8 (5.3)	9.7 (5.0)	12.3 (6.9)	8.4 (2.7)
C-reactive protein, mg/L, mean (SD) [median; Q1, Q3]	10.8 (12.4) [7.4; 2.2, 13.3]	9.5 (20.4) [3.6; 1.9, 7.6]	9.2 (11.8) [5.9; 4.0, 8.0]	5.1 (6.1) [3.2; 0.8, 5.5]
MDGA (10-cm VAS)	58.4 (17.6)	54.1 (18.0)	57.0 (14.5)	58.1 (18.7)
PtGA (10-cm VAS)	60.6 (23.6)	48.0 (23.8)	62.2 (16.8)	53.8 (27.7)
Week 24				
DAS 28	3.4 (1.2)	3.5 (1.5)	4.2 (1.2)	3.8 (1.4)
CDAI, mean (SD) [median; Q1, Q3]	12.4 (9.6) [10.7; 5.1, 19.4]	12.4 (10.7) [11.1; 3.4, 20.7]	16.4 (10.8) [15.6; 6.8, 25.4]	17.7 (12.8) [18.1; 8.6, 28.1]
Tender joint count (28 joints)	3.4 (4.3)	4.2 (5.6)	6.5 (4.9)	5.3 (6.7)
Swollen joint count (28 joints)	3.8 (3.6)	2.8 (3.3)	4.8 (6.5)	4.5 (4.6)
Tender joint count (68 joints)	5.4 (8.1)	7.4 (10.5)	9.8 (9.3)	7.2 (8.3)
Swollen joint count (66 joints)	4.6 (4.6)	3.9 (4.6)	5.7 (8.0)	6.2 (6.2)
CRP, mg/L, mean (SD) [median; Q1, Q3]	5.0 (5.4) [3.3; 1.8, 5.6]	6.0 (6.5) [2.7; 1.9, 8.5]	13.2 (10.6) [10.1; 3.4, 20.6]	3.6 (4.0) [2.3; 0.6, 3.6]
ACR20 response, *n* (%)	19 (61.3%)	19 (65.5%)	7 (70.0%)	4 (30.8%)
ACR50 response, *n* (%)	10 (32.3%)	11 (37.9%)	3 (30.0%)	2 (15.4%)
ACR70 response, *n* (%)	6 (19.4%)	7 (24.1%)	1 (10.0%)	1 (7.7%)
MDGA (100-mm VAS)	26.8 (19.6)	27.3 (22.1)	23.7 (19.2)	39.3 (25.9)
PtGA (100-mm VAS)	25.5 (21.8)	28.0 (24.3)	27.1 (22.3)	41.8 (27.4)
FACIT Fatigue	73.8 (19.9)	74.0 (18.9)	64.4 (22.8)	62.1 (22.8)
HAQ-DI	1.0 (0.6)	0.9 (0.48)	1.1 (0.5)	1.1 (0.3)
SF-36 Physical functioning Physical role limitations Pain General health perceptions Energy/vitality Social functioning Emotional role limitations Mental health	68.2 (23.5) 63.8 (43.1) 58.3 (21.0) 56.8 (14.5) 62.2 (20.1) 81.3 (23.4) 72.4 (41.9) 67.4 (18.6)	63.0 (27.4) 57.6 (44.9) 62.7 (23.5) 50.3 (21.1) 56.9 (20.2) 81.8 (19.9) 66.7 (42.4) 67.1 (19.1)	52.0 (25.3) 59.4 (49.9) 55.9 (22.2) 52.5 (22.2) 51.5 (22.5) 71.3 (26.3) 50.0 (47.2) 63.2 (20.6)	48.5 (19.2) 36.5 (42.8) 47.5 (20.4) 41.5 (19.9) 43.5 (17.5) 65.5 (20.4) 43.6 (49.8) 61.2 (18.2)

^a^ Anti-CCP levels ≥ 7 relative units/mL. ^b^ In patients who reported corticosteroid use at baseline (*n* = 25, 18, 4, and 12 for rituximab→rituximab 1000 mg, rituximab→rituximab 500 mg, placebo→rituximab 1000 mg, and placebo→rituximab 1000 mg groups, respectively). ^c^ Because patients were required to receive stable doses of therapies, the data shown here also apply to week 24. ACR, American College of Rheumatology; BMI, body mass index; CCP, cyclic citrullinated peptide; CRP, C-reactive protein; DAS28, Disease Activity Score–28 joints; DMARD, disease-modifying antirheumatic drug; FACIT, Functional Assessment of Chronic Illness Therapy; HAQ-DI, Health Assessment Questionnaire-Disability Index; MDGA, physician global activity; PtGA, patient global activity; Q, quartile; RF, rheumatoid factor; SF-36, Short-form 36; TNF, tumor necrosis factor; VAS, visual analogue scale.

**Table 2:** Adverse events and serious adverse events during Part 2 (week 24–52) of the AMARA study by system organ class. Patients could have more than one adverse event or serious adverse event.

Adverse Events by SOC	Rituximab→Rituximab		Placebo→Rituximab		Total (*n* = 83)
	Rituximab 1000 mg (*n* = 31)	Rituximab 500 mg (*n* = 29)	Rituximab 1000 mg (*n* = 10)	Rituximab 500 mg (*n* = 13)	
AEs ≥5% in any subgroup					
Number of AEs	38	33	7	17	95
Number (%) of patients	26 (83.9%)	26 (89.7%)	7 (70.0%)	12 (92.3%)	71 (74.7%)
Infections and infestations	19 (61.3%)	14 (48.3%)	3 (30.0%)	6 (35.3%)	42 (50.6%)
Skin and subcutaneous tissue disorders	3 (9.7%)	5 (17.2%)	1 (10.0%)	1 (7.7%)	10 (12.0%)
Musculoskeletal and connective tissue disorders	2 (6.5%)	2 (6.9%)	1 (10.0%)	2 (15.4%)	7 (8.4%)
Investigations	2 (6.5%)	2 (6.9%)	0	1 (7.7%)	5 (6.0%)
Gastrointestinal disorders	3 (9.7%)	1 (3.4%)	0	0	4 (4.8%)
General disorders and administration site conditions	1 (3.2%)	1 (3.4%)	0	2 (15.4%)	4 (4.8%)
Nervous system disorders	2 (6.5%)	1 (3.4%)	0	1 (7.7%)	4 (4.8%)
Ear and labyrinth disorders	1 (3.2%)	0	1 (10.0%)	1 (7.7%)	3 (3.6%)
Surgical and medical procedures	0	2 (6.9%)	0	1 (7.7%)	3 (3.6%)
Metabolism and nutrition disorders	0	1 (3.4%)	1 (10.0%)	0	2 (2.4%)
Vascular disorders	1 (3.2%)	0	0	1 (7.7%)	2 (2.4%)
Immune system disorders	0	0	0	1 (7.7%)	1 (1.2%)
SAEs (all)					
Number of events	6	7	1	3	17
Number (%) of patients	5 (16.1%)	7 (24.1%)	1 (10.0%)	3 (23.1%)	16 (19.3%)
Surgical and medical procedures ^a^	1 (3.2%)	3 (10.3%)		1 (7.7%)	5 (6.0%)
Infections and infestations ^b^	2 (6.5%)	1 (3.4%)	0	1 (7.7%)	4 (4.8%)
Musculoskeletal and connective tissue disorders ^c^	1 (3.2%)	2 (6.9%)	1 (10.0%)	0	4 (4.8%)
General disorders and administration site conditions ^d^	1 (3.2%)	0	0	1 (7.7%)	2 (2.4%)
Gastrointestinal disorders ^e^	1 (3.2%)	0	0	0	1 (1.2%)
Vascular disorders ^f^	0	1 (3.4%)	0	0	1 (1.2%)

^a^ Preferred term of bunion operation, invertebral disc operation (2), nasal operation, and joint arthroplasty. ^b^ Preferred terms of abdominal wall abscess, respiratory tract infection, gastroenteritis, and erysipelas. ^c^ Preferred terms of muscular weakness, arthropathy, invertebral disc protrusion, and osteoarthritis. ^d^ Preferred term of pyrexia. ^e^ Preferred term of pancreatitis. ^f^ Preferred term of hypertensive crisis. AE, adverse events; SAE, serious adverse events; SOC, system organ class.

## Data Availability

The data presented in this study are available for collaborative research from the corresponding author on reasonable request.

## Supplementary Materials

The following supporting information can be downloaded at: https://www.mdpi.com/article/10.3390/jcm11247316/s1, Section S1: Study investigators who enrolled patients in the AMARA study; Figure S1: Changes in B cell counts from week 24 until week 52 of the AMARA study.
