# Supplementary material for: Effectiveness of Different Rituximab Doses Combined with Leflunomide in the Treatment or Retreatment of Rheumatoid Arthritis: Part 2 of a Randomized, Placebo-Controlled, Investigator-Initiated Clinical Trial (AMARA)
Source: J Clin Med. 2022 Dec 9;11(24):7316. doi: 10.3390/jcm11247316 (PMC9784147; doi:10.3390/jcm11247316)
Supplement: Supplementary file 1 [file jcm-11-07316-s001.zip › jcm-2045849-supplementary.pdf]

Supplementary Material

Effectiveness of different doses of rituximab in combination with leflunomide in the treatment or retreatment of rheumatoid arthritis: part 2 of a randomized, placebo-controlled, investigator-initiated clinical trial (AMARA study)

Supplementary Section S1. Study investigators who enrolled patients in the AMARA study

Baden-Baden: Prof. Christoph Fiehn

Bad Kösen: Dr. Jörg Kaufmann

Bad Nauheim: Prof. Ulf Müller-Ladner

Berlin: Dr. Rieke Alten, Prof. Marina Backhaus, Prof. Gerd-Rüdiger Burmester, Prof. Andreas Krause, Dr. Sven Remstedt

Dresden: Dr. Leonore Unger

Erlangen: Dr. Jörg Wendler

Frankfurt am Main: Prof. Harald Burkhardt

Freiburg: Dr. Bettina Bannert

Goslar: Dr. Karin Rockwitz

Göttingen: Prof. Sabine Blaschke

Greifswald: Dr. Michael Fiene

Hedielberg: Dr. Bernhard Heilig

Hofheim am Taunus: Dr. Lothar Meier

Homburg: Dr. Gunter Assmann

Köln: Prof. Andrea Rubbert-Roth

Leipzig: Prof. Christoph Baerwald

Ludwigsfelde: Dr. Jörg Kaufmann

Mainz: Prof. Peter Härle

München: Prof. Herbert Kellner, Prof. Klaus Krüger

Osnabrück: Dr. Georg Gauler

Pima: Dr. Anett Grässler

Planegg: Dr. Martin Welcker

Ratingen: Dr. Siegfried Wassenberg

Regensburg: Prof Martin Fleck

Trier: Prof. Stefan Weiner

Tübingen: Dr. Joerg Henes

Ulm: Dr. Andreas Viardot

Wuppertal: Dr. Carl-Johannes Heinmüller

Würzburg: Prof. Hans-Peter Tony

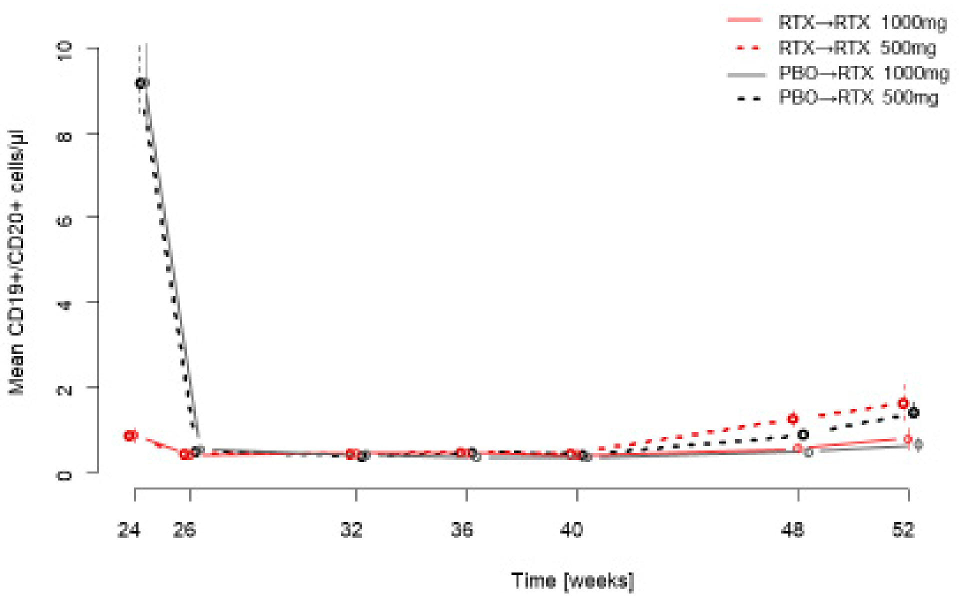

Supplementary Figure S1. Changes in B cell counts from week 24 until week 52 of the AMARA study. *PBO*, placebo; *RTX*, rituximab.
